# Green-synthesized silver nanoparticles from edible plant extracts ameliorate cadmium chloride-induced hepatorenal and testicular toxicity in rats

**DOI:** 10.3389/fcell.2025.1677434

**Published:** 2025-12-01

**Authors:** Akram Abu-Okail, Abdullah S. M. Aljohani, Ayman M. Mousa, Heba A. S. El-Nashar, Mohamed El-Shazly, Ramy K. A. Sayed, Waleed Al Abdulmonem, Mohamed Farghali, Heba F. Kamaly, Ibrahim M. El-Ashmawy, Ahmed A. H. Abdellatif, Nashwa Hamad

**Affiliations:** 1 Department of Pathology and Laboratory Diagnosis, College of Veterinary Medicine, Qassim University, Buraydah, Saudi Arabia; 2 Department of Medical Biosciences, College of Veterinary Medicine, Qassim University, Buraydah, Saudi Arabia; 3 Department of Basic Health Sciences, College of Applied Medical Sciences, Qassim University, Buraidah, Saudi Arabia; 4 Department of Pharmacognosy, Faculty of Pharmacy, Ain Shams University, Cairo, Egypt; 5 Department of Anatomy and Embryology, Faculty of Veterinary Medicine, Sohag University, Sohag, Egypt; 6 Department of Pathology, College of Medicine, Qassim University, Buraydah, Saudi Arabia; 7 Department of Animal and Poultry Hygiene and Environmental Sanitation, Faculty of Veterinary Medicine, Assiut University, Assiut, Egypt; 8 Department of Forensic Medicine and Toxicology, Faculty of Veterinary Medicine, Assiut University, Assiut, Egypt; 9 Department of Pharmaceutics, College of Pharmacy, Qassim University, Buraydah, Saudi Arabia; 10 Department of Pathology, Faculty of Veterinary Medicine, Assiut University, Assiut, Egypt

**Keywords:** green-silver nanoparticles, cadmium, antioxidant, liver, kidney, testes

## Abstract

**Background:**

Cadmium (Cd) is widely known as an environmental toxicant, ranked as the seventh most toxic heavy metal. Exposure to cadmium through inhalation and ingestion can lead to serious health issues, including liver damage, kidney degeneration, testicular problems, and blood disorders in both humans and animals.

**Objective:**

This study aimed to evaluate the protective effects of biosynthesized silver nanoparticles (AgNPs) against cadmium chloride-induced hepatic, renal, and reproductive toxicity in male rats.

**Methods:**

AgNPs were synthesized via chemical reduction in silver nitrate using a combination of three plant extracts, namely, *Petroselinum crispum*, *Zea mays* silk, and *Acacia senegal*. The obtained AgNPs were characterized and subjected to an *in vivo* study. Forty healthy adult male albino rats (200–230 g) were divided into four groups (n = 10): G1: negative control, G2: rats received AgNPs (200 mg/kg b.w), G3: rats received cadmium chloride (5 mg/kg b.w), and G4: rats received AgNPs (200 mg/kg b.w.) followed by cadmium chloride (5 mg/kg b.w) after 90 min. All treatments were administered daily for 35 days. Biochemical assessments included liver enzymes (alanine transaminase, aspartate aminotransferase, and alkaline phosphatase), kidney markers (urea and creatinine), testicular hormones (testosterone, luteinizing hormone, and follicle-stimulating hormone), lipid profile (low-density lipoprotein, high-density lipoprotein, and triglyceride), and antioxidant markers (total antioxidants and malondialdehyde). Histopathological studies were performed on the liver, kidney, and testicular tissues.

**Results:**

Synthesized AgNPs exhibited spherical morphology, with an average nanosize distribution of 5.28–21.47 nm. Cadmium chloride exposure significantly elevated liver enzymes, lipid markers, urea, creatinine, and MDA while decreasing testicular hormone levels (testosterone and luteinizing hormone), indicating hepato-renal and testicular damage, alongside histopathological damage in all examined organs. Co-administration of AgNPs markedly ameliorated these biochemical alterations, improving liver and kidney function, restoring total antioxidant capacity, and normalizing lipid, protein, and testicular hormone profiles. Histopathological results revealed that treatment with AgNPs restored the angiopathic, degenerative, and necrotic changes prompted by cadmium chloride administration.

**Conclusion:**

AgNPs biosynthesized from combined extracts of *P. crispum*, *Z. mays* silk, and *A. senegal* demonstrated significant protective effects against cadmium chloride-induced toxicity. Their antioxidant and free radical scavenging properties suggest potential therapeutic value in mitigating environmental cadmium toxicity.

## 1 Introduction

Nanotechnology is an extensively growing field that involves the synthesis and characterization of noble metals such as silver, gold, and platinum as nanoparticles (Helmy et al., 2020a). These nanoparticles have attracted considerable attention in diverse applications, including drug delivery, bioengineering, textile engineering, biological labeling, biotechnology, catalysis, water treatment, and the detection of genetic disorders (Mollick et al., 2019). Their unique properties, determined by size, shape, and atomic distribution, make them particularly valuable (Shawkey et al., 2013). Different methods are used to synthesize nanoparticles, such as heat evaporation (Bae et al., 2002), chemical reduction (Maity et al., 2011), photochemical (Callegari et al., 2003), electrochemical (Yin et al., 2003), thermal decomposition (Jen-La Plante et al., 2010), radiation (Dimitrijevic et al., 2001), and microwave-assisted methods (Anis et al., 2023). However, these approaches often require high energy input and pose hazardous effects (Helmy et al., 2020a). To address these drawbacks, biological systems such as plants, bacteria, and fungi have emerged as sustainable and eco-friendly alternatives for nanoparticle biosynthesis (Donda et al., 2013).

Plants are particularly promising as they contain bioactive compounds such as alkaloids, terpenoids, flavonoids, and tannins that act as natural reducing and stabilizing agents in nanoparticle formation (Burketová et al., 2022). Antioxidant-rich plants, in particular, have shown strong potential for producing biologically active nanoparticles (Mustapha et al., 2022). Utilizing local plants, plant exudates, and plant waste products for nanoparticle synthesis offers the dual advantages of minimal cost and reduced environmental and human health risks (Alharbi et al., 2022).

In this study, three edible plants with high antioxidant properties, namely, *Petroselinum crispum* (parsley), *Zea mays* L. (corn silk), and *Acacia senegal* L., were selected for the green synthesis of silver nanoparticles (AgNPs). Parsley leaves, abundant in flavonoids and ascorbic acid, provide potent reducing agents for nanoparticle synthesis (Wang et al., 2022). Zea mays (corn) silver nanoparticles exhibited potent antioxidant capacity, as verified by 2,2-diphenyl-1-picrylhydrazyl (DPPH) radical scavenging, 2,2′-azino-bis(3-ethylbenzothiazoline-6-sulfonic acid (ABTS) radical scavenging, nitric oxide scavenging, and reducing power assays (Singh et al., 2022). Additionally, *Acacia senegal* gums, rich in polysaccharides, not only reduce and stabilize AgNPs but also prevent aggregation and enhance their biological activity (Ali et al., 2020).

Cadmium (Cd) is widely known as an environmental toxicant, classified by the World Health Organization as a priority food pollutant and a widespread endocrine-disrupting chemical (Wang W. et al., 2012). The exposure to Cd through inhalation and ingestion can cause acute and chronic toxic manifestations (Al-Anazi et al., 2015). It has been extensively reported that cadmium toxicity damages liver cells, degenerates renal proximal tubules, impairs the testes, and causes erythrocyte disorders in both humans and animals (Kara et al., 2007). The reproductive system is particularly vulnerable to cadmium toxicity, often resulting in reduced male fertility, low sperm count, and poor semen quality (Siu et al., 2009). The primary mechanism underlying Cd toxicity is cellular oxidative damage, which induces lipid peroxidation in the membranes of organs where cadmium accumulates (Stohs et al., 2001). Cd exposure decreases essential antioxidants such as glutathione (GSH) and protein-binding sulfhydryl groups, leading to an overproduction of reactive oxygen species (ROS), including hydrogen peroxide, hydroxyl radicals, and superoxide ions. This oxidative stress causes lipid peroxidation, disrupts intracellular stability, damages DNA and cell membranes, alters gene expression, and ultimately induces cell death (Stohs et al., 2001).

In several studies, lipid peroxidation has been reported in acute or chronic cadmium poisoning, resulting in elevated tissue malondialdehyde (MDA) levels, depletion of GSH-Px, and changes in several enzymes, such as SOD, alanine transaminase (ALT), aspartate aminotransferase (AST), blood urea nitrogen, and serum creatinine levels (El-Demerdash et al., 2004). Cd also causes testicular vascular damage, lowers oxygen supply to seminiferous tubules, increases adrenaline and noradrenaline production and sympathetic nerve stimulation, and induces vasoconstriction (Areba, 2020). Current treatment strategies typically involve chelating agents and antioxidant therapy to reduce Cd’s toxic impacts (Jurczuk et al., 2004).

Our study aimed to evaluate the effects of green-synthesized AgNPs from a combination of three edible plant extracts, namely, *P. crispum*, *Z. mays L.*, *and Acacia Senegal (L.)*, as a prophylactic agent against cadmium-induced toxicity in male albino rats.

## 2 Materials and methods

### 2.1 Chemicals and reagents

Cadmium chloride (CdCl_2_) was purchased from Sigma-Aldrich (Germany). ELISA kits for testosterone, luteinizing hormone (LH), and follicle-stimulating hormone (FSH), along with liver function tests [alanine transaminase (ALT), aspartate aminotransferase (AST), alkaline phosphatase (ALP), and bilirubin] and kidney function tests (urea and creatinine), were used according to the manufacturer’s instructions. Formalin solution (10%, Sigma-Aldrich, Germany), ethyl alcohol (95%; Saudi Pharmaceutical Industries, Saudi Arabia), xylol (Sigma-Aldrich, Germany), and hard paraffin (Chemact [Liaoning] Petrochemicals Ltd., China) were used for tissue processing. Mayer’s hematoxylin and eosin stains were purchased from Abcam (United States).

### 2.2 Plant materials and extraction

*Petroselinum crispum* and *A. senegal* were purchased from the local market in Qassim, Saudi Arabia. *Zea mays* silk was collected during the flowering season (February–May) from the natural pastures and lands surrounding the farms in Qassim, Saudi Arabia. The plants were dried well in the air, and all parts of the plant (paper, roots, sticks, and flowers) were ground to obtain a very fine powder from the plant. Two liters of methanol (99.9%) were added to 200 g of the powder, agitated, left for 72 h, and then filtered, and the filtrate was kept in a clean and dry flask. The methanol extracts were concentrated using a “rotary evaporator,” and the diluted suspension was preserved in the form of a paste in a refrigerator at −12 °C until use.

### 2.3 Synthesis of silver nanoparticles

Approximately 5 g of each extract was added to 100 mL of distilled water and heated in a water bath at 60 °C for 30 min. For *Acacia senegal*, the heating time was extended to 60 min to deactivate the oxidase enzyme. The resulting decoction was then filtered using Whatman No. 1 filter paper. The filtrate (aqueous extract) was used as a control and for subsequent characterization and biological studies. Silver nanoparticles were synthesized by mixing different aqueous extracts with 1 mM (0.001 M) silver nitrate in a ratio of 2:10, followed by the addition of two drops of 1 N NaOH, and kept in a water bath for 10 min at 60 °C.

### 2.4 Characterization of synthesized silver nanoparticles

The colloidal solutions of synthesized AgNPs were monitored for visual color change. The size in nm of prepared AgNPs was measured and identified using differential light scattering (DLS) (Zetasizer-Nano90; Malvern Instrument GmbH, Germany). The average of three measured samples without dilutions was recorded. The laser beam and angle of the instrument were 623 nm and 90°, respectively, at 35 °C. Furthermore, the AgNPs were subjected to scanning electron microscopy (SEM) (FESEM, supra 55–Carl Zeiss, Germany) for determining the morphology of the prepared AgNPs (Abdellatif, 2020; Abdellatif et al., 2020a; Abdellatif et al., 2020b). Furthermore, the features (size and morphology) of the synthesized AgNPs were characterized using transmission electron microscopy (TEM) by following standard procedures in the literature (Helmy et al., 2020b). For TEM analysis, a drop of the AgNP suspension was placed on a carbon-coated copper grid and dried under ambient conditions for 10 min. The particle shape, size, and distribution were then evaluated using TEM (JEOL JEM-100 CXII, Tokyo, Japan) operated at an acceleration voltage of 120 kV.

### 2.5 Animals

Forty healthy adult male albino rats (200–230 g body weight) were obtained from the Faculty of Pharmacy, King Saud University, Riyadh, Kingdom of Saudi Arabia. Rats were housed in cages and fed laboratory animal feed pellets from the Saudi Grains Organization (SAGO), with water provided for a period of 2 weeks before the beginning of the experiment to ensure healthy conditions and to exclude emaciated animals. The study is ethically approved by the Qassim University Ethical Committee (QUEC) (approval number: (3/1)1443-1444H).

### 2.6 Study design

**FIGURE 1:**
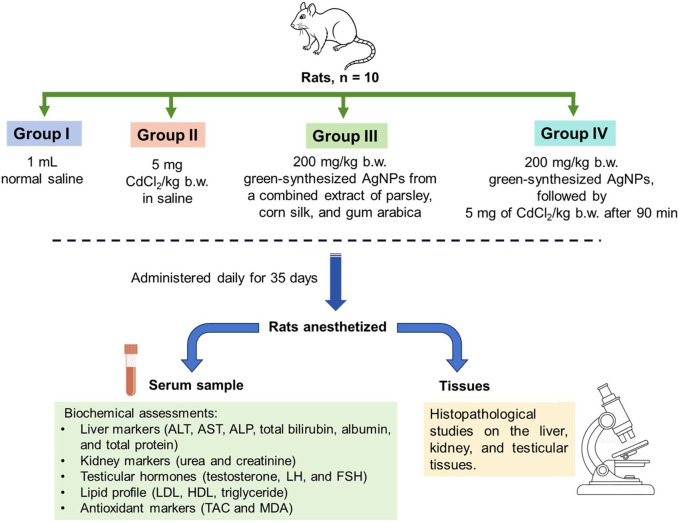
Illustration depicting a treatment protocol for CdCl_2_-intoxicated rats.

The study design is illustrated in Figure 1. The rats were randomly divided into four equal groups (n = 10) and received the following treatments orally using intragastric tubes once daily for 35 days (complete spermatogenic cycle) as follows:

Group I: Control rats received 1 mL of normal saline (Helmy et al., 2020a).
Group II: Rats received 5 mg of CdCl_2_/kg b.w. in a final volume of 1 mL saline of the oral LD_50_ values in rats (Bashir et al., 2019).
Group III: Rats received 200 mg/kg b.w. of synthesized silver nanoparticles from a combined extract of parsley, corn silk, and gum arabica (Helmy et al., 2020b).
Group IV: Rats received 200 mg/kg b.w. of synthesized silver nanoparticles from a combined extract of parsley, corn silk, and gum arabica and were then administered 5 mg/kg body weight of CdCl_2_ in saline to in a final volume of 1 mL saline at an interval of 90 min (Bashir et al., 2019).

The rats’ food intake, water consumption, body weight, and overall health status were regularly monitored throughout the experiment to ensure proper animal welfare and the reliability of the experimental outcomes. At the end of the experiment, the rats were anesthetized by inhalation of diethyl ether; then, an intracardiac blood sample was taken and preserved at 4 °C for 30 min. The collected blood sample was centrifuged at 4,000 rpm for 10 min to obtain serum samples, which were kept frozen at −80 °C. Targeted tissues (liver, kidney, and testes) were collected and fixed in 10% neutral buffered formalin. Furthermore, to prevent any potential experimental bias, the investigators were blinded throughout all stages of the study, including sample collection, processing, and data analysis.

### 2.7 Assessment of liver function tests

Liver enzymes, including ALT, AST, and ALP, were measured spectrophotometrically using ready-to-use kits and according to the manufacturer’s instructions for the assessment of liver function and damage, along with the levels of total bilirubin, albumin, and total protein.

### 2.8 Assessment of kidney function tests

Kidney function tests (serum urea and serum creatinine) were measured spectrophotometrically using commercial kits, according to the manufacturer’s instructions.

### 2.9 Assessment of reproductive system functions

Testosterone, LH, and FSH levels were determined according to previously described methods (Salem and Salem, 2016; Wang et al., 2017).

### 2.10 Assessment of oxidative stress and lipid markers

Oxidative stress status was evaluated by determining total antioxidants (TAC) and malondialdehyde (MDA) levels using commercial kits, according to the manufacturer’s instructions (Elmallah et al., 2017; Kini et al., 2018; Padma et al., 2016). Lipid profile [triglycerides (TGs), low-density lipoprotein (LDL), and high-density lipoprotein (HDL)] was also analyzed using commercial kits.

### 2.11 Histopathological studies

For qualitative analysis of liver, kidney, and testis histology, the samples were fixed, dehydrated, cleared, and embedded in paraffin. Sections of tissue (4 μm thick) were prepared using a rotary microtome and stained with hematoxylin and eosin stain for microscopic observations (Eissa et al., 2025). Stained sections were examined using a light microscope and photographed using a digital camera.

### 2.12 Statistical analysis

All biochemical indicators, including liver function (ALT, AST, ALP, albumin, total protein, and bilirubin), kidney function (urea and creatinine), reproductive hormones (testosterone, LH, and FSH), lipid profile (TGs, LDL, and HDL), and oxidative stress markers (TAC and MDA), were statistically analyzed using Minitab software (version 20, Minitab Inc., State College, PA, United States). One-way analysis of variance (ANOVA) was used to assess the effects of CdCl_2_ toxicity and the protective role of silver nanoparticles compared to the untreated control. Post hoc comparisons between groups were performed using Tukey’s test, with statistical significance set at *p* < 0.05. Data are presented as the mean ± standard deviation.

## 3 Results

The obtained AgNPs were prepared by the reduction of different plant extracts, including *P. crispum* (parsley*), Z. mays* L. (corn silk), and *Acacia senegal* (gum acacia). The obtained AgNPs were of nanosize and had no aggregations. The colloidal solutions of the synthesized AgNPs were monitored for a visual color change to brown.

### 3.1 Characterization of synthesized AgNPs

**FIGURE 2:**
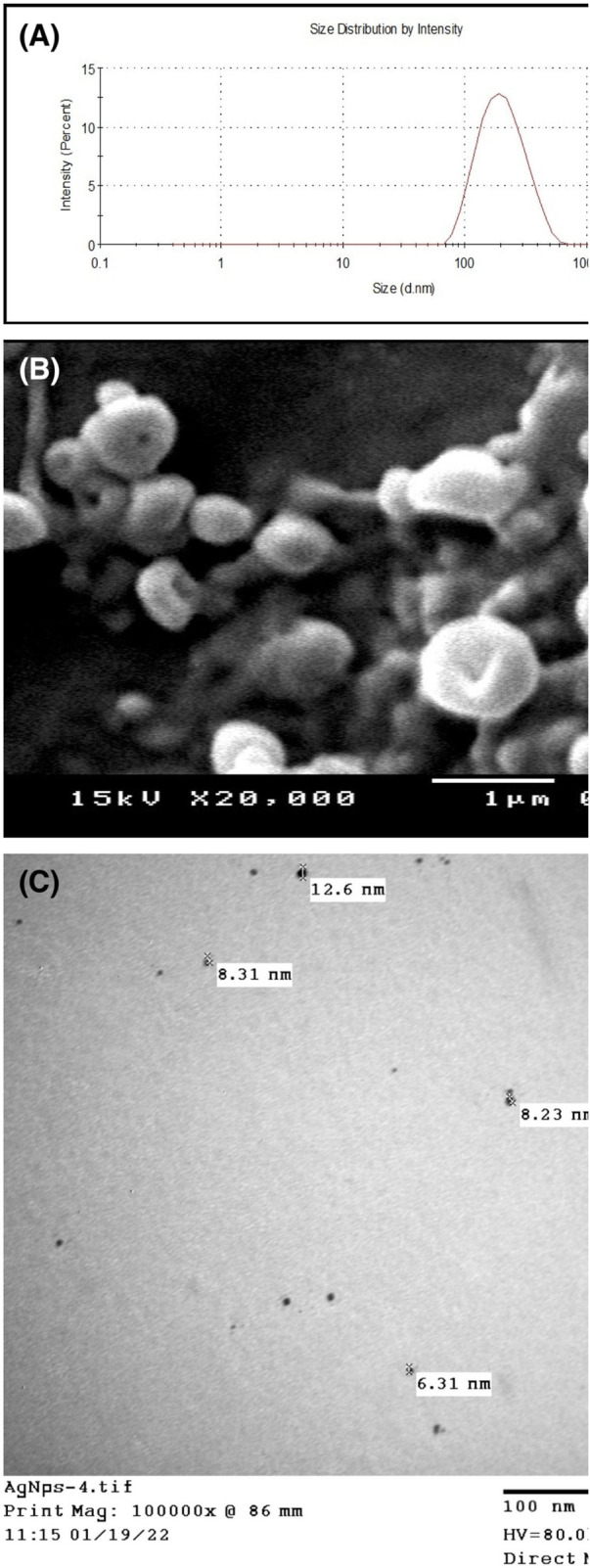
Characterizations of AgNPs using dynamic light scattering **(A)**, scanning electron microscope **(B)**, and transmission electron microscope **(C)**; the nanoparticles were in average nanosize and have no aggregations.

The DLS analysis verified symmetrical peaks of AgNPs with an average size of 495 ± 20.1 nm (Figure 2A). All recorded polydispersity indices (PDIs) were 0.112–0.145, which were below the accepted range, signifying a stable colloidal system. Moreover, the data obtained from SEM showed spherical AgNPs, along with some cubic-shaped particles, with an average diameter of 1 µm (Figure 2B). As demonstrated in Figure 2C, TEM analysis showed a range of sizes, between 5.28 and 23.7 nm. TEM suggested that the morphology of the synthesized nanoparticles is spherical, with a mean size in the nano-range, indicating the successful synthesis of AgNPs.

### 3.2 Biochemical analysis

#### 3.2.1 Effect of synthesized AgNPs on the liver functions

The effects of different treatments on the liver enzymes are illustrated in Table 1. The results showed a significant elevation of AST, ALT, and ALP activities, along with total bilirubin levels by 2.27-fold (*p* < 0.0001), 4.08-fold (*p* < 0.0001), 3.85-fold (*p* < 0.0001), and 1.89-fold (*p* = 0.16347), respectively, in the CdCl_2_-treated group, compared with those in the control healthy group. Meanwhile, the levels of albumin and total protein were decreased by 1.58-fold (*p* = 0.0008) and 1.61-fold (*p* < 0.002), respectively, in the CdCl_2_-treated group. The AgNP-treated rats demonstrated normal activities of liver enzymes, close to the values of the control group, indicating non-toxic effects of the extract on the liver. However, treatment of CdCl_2_-intoxicated rats with AgNPs induced a dramatic decrease in the elevated activities of ALT, AST, ALP, and total bilirubin levels by 64.61% (*p* < 0.0001), 50.87% (*p* < 0.0001), 64.62% (*p* < 0.0001), and 30.15% (*p* = 0.509), respectively, along with an increase in albumin (+44.14%, *p* = 0.009) and total protein (+42.73%, *p* = 0.04), compared to those in the CdCl_2_ group, suggesting the hepatoprotective role of the AgNPs.

**TABLE 1:** Effect of different treatments on the liver function tests.

Group	ALT (U/L)	AST (U/L)	ALP (U/L)	Albumin (g/dL)	Total protein (g/dL)	Total bilirubin (g/dL)
Control	41.58 ± 7.36^a^	127.17 ± 17.34^b^	80.48 ± 13.95^b^	4.57 ± 0.70^a^	8.87 ± 1.79^a^	0.72 ± 0.31^a^
AgNP	45.50 ± 6.75^a^	133.50 ± 13.56^b^	92.37 ± 14.41^b^	4.33 ± 0.86^a^	8.55 ± 1.34^a^	0.76 ± 0.61^a^
CdCl_2_	170.00 ± 17.98^b^	289.00 ± 59.62^a^	309.70 ± 5.06^a^	2.90 ± 0.40^b^	5.50 ± 0.11^b^	1.36 ± 0.68^a^
AgNP + CdCl_2_	60.17 ± 14.44^a^ (−64.61%)	142.00 ± 20.13^b^ (−50.9%)	109.58 ± 17.83^b^ (−64.62%)	4.18 ± 0.38^a^ (+44.14%)	7.85 ± 1.68^a^ (+42.73%)	0.95 ± 0.34^a^ (−30.15%)

Values expressed as the mean ± SD of six replicates. Means within the same column that do not share a common superscript letter (a–b) differ significantly at p < 0.05, n=6. Significant differences: CdCl_2_ vs. control (*p* < 0.0001 for ALT, AST, and ALP; *p* = 0.0008, 0.00245 for albumin and protein), AgNPs vs. CdCl_2_ (*p* < 0.0001 for ALT, AST, and ALP; *p* = 0.00347 and 0.00596 for albumin and protein, respectively), and AgNPs + CdCl_2_ vs. CdCl_2_ (*p* < 0.0001 for ALT, AST, and ALP; *p* = 0.00896 and 0.03962 for albumin and protein, respectively). Percentage of change between brackets = [(M_AgNPs+CdCl2_−M_CdCl2)_/M_CdCl2_] × 100.

#### 3.2.2 Effect of synthesized AgNPs on the kidney functions

The data presented in Table 2 demonstrate that the creatinine and urea levels were not significantly changed (*p* > 0.05) in rats that received AgNPs only, compared with the healthy control group. Meanwhile, the creatinine and urea levels were significantly increased (*p* < 0.01) in CdCl_2_ rats compared with the healthy control group. The treatment of CdCl_2_ rats with synthesized AgNPs decreased the creatinine and urea levels by 18.05%% (*p* > 0.05) and 58.14% (*p* < 0.05), respectively, compared with those in the CdCl_2_ group.

**TABLE 2:** Effect of different treatments on the kidney function tests.

Group	Urea level (mg/dL)	Creatinine level (mg/dL)
Control	39.33 ± 13.28^b^	0.42 ± 0.26^b^
AgNP	48.17 ± 21.25^b^	0.45 ± 0.14^b^
CdCl_2_	80.33 ± 7.47^a^	1.29 ± 0.63^a^
AgNP + CdCl_2_	65.83 ± 19.82^ab^ (−18.05%)	0.54 ± 0.42^b^ (−58.14%)

Values expressed as the mean ± SD of six replicates. Means within the same column that do not share a common superscript letter (a–b) differ significantly at *p* < 0.05, n = 6. Significant differences: CdCl_2_ vs. control (*p* = 0.0017 and 0.00767), AgNPs vs. CdCl_2_ (*p* = 0.01402 and 0.00958), and AgNPs + CdCl_2_ vs. CdCl_2_ (*p* = 0.4388 and 0.02357) for urea and creatinine, respectively. Percentage of change between brackets = [(M_AgNPs+CdCl2_−M_CdCl2)_/M_CdCl2_] × 100.

#### 3.2.3 Effect of synthesized AgNPs on the testicular hormones

The effects of different treatments on testicular hormones, including testosterone, LH, and FSH, are listed in Table 3. The results showed a significant decrease in the levels of testosterone and LH by 2.08-fold (*p* < 0.05) and 3.71-fold (*p* < 0.05), respectively, in the CdCl_2_-treated group compared to control rats, while FSH showed a non-significant decrease of 2.16-fold (*p* > 0.05), compared to that in control rats. In contrast, AgNP-treated rats demonstrated normal levels of testicular hormones compared to those of the control group, indicating that the extract has non-toxic effects on the testes. However, treatment of CdCl_2_-intoxicated rats with AgNPs induced a marked increase in the levels of testosterone, LH, and FSH by 91.53%, 130.77%, and 128.75%, respectively, compared to those in the CdCl_2_ group, suggesting the protective effect of the AgNPs toward the toxicity of CdCl_2_ on the reproductive functions.

**TABLE 3:** Effect of different treatments on the levels of testicular hormones.

Group	Testosterone level (ng/mL)	LH level (mIU/mL)	FSH level (mIU/mL)
Control	2.46 ± 0.43^a^	3.86 ± 1.35^a^	3.46 ± 2.01^a^
AgNP	2.28 ± 0.73^a^	3.64 ± 1.93^a^	3.82 ± 2.01^a^
CdCl_2_	1.18 ± 0.66^b^	1.04 ± 0.65^b^	1.60 ± 0.63^a^
AgNP + CdCl_2_	2.26 ± 0.40^a^ (+91.53%)^#^	2.40 ± 1.27^ab^ (+130.77%)^#^	3.66 ± 2.07^a^ (+128.75%)^#^

Values expressed as the mean ± SD of six replicates. Means within the same column that do not share a common superscript letter (a–b) differ significantly at *p* < 0.05, n = 6. Significant differences were found in CdCl_2_ vs. control (testosterone, *p* = 0.01314; LH, *p* = 0.02401), AgNPs vs. CdCl_2_ (testosterone, *p* = 0.0354; LH, *p* = 0.03946), and AgNPs + CdCl_2_ vs. CdCl_2_ (testosterone, *p* = 0.03943). No significant differences for FSH (all *p* > 0.05).

^#^Percentage of change between brackets = [(M_AgNPs+CdCl2_−M_CdCl2)_/M_CdCl2_] × 100.

#### 3.2.4 Effect of synthesized AgNPs on the lipid profile

The effects of different treatments on the lipid profile, including LDL, HDL, and TGs, are displayed in Table 4. The results showed a significant elevation in the levels of LDL and TG (*p* < 0.0001) in the CdCl_2_ group by 3.30- and 2.41-fold, respectively, compared to those in the control group. Meanwhile, HDL was significantly decreased by 2.24-fold in the CdCl_2_ group (*p* < 0.01).

**TABLE 4:** Influence of different treatments on the lipid profile.

Group	LDL level (mg/dL)	HDL level (mg/dL)	Triglyceride level (mg/dL)
Control	17.77 ± 3.01^c^	55.10 ± 9.04^a^	32.63 ± 8.24^b^
AgNP	24.53 ± 5.38^bc^	54.63 ± 16.50^a^	35.32 ± 8.63^b^
CdCl_2_	58.65 ± 6.03^a^	24.57 ± 6.55^b^	78.55 ± 10.40^a^
AgNP + CdCl_2_	27.72 ± 4.60^b^ (−52.74%)^#^	52.72 ± 15.15^a^ (+114.57%)^#^	36.53 ± 11.01^b^ (−53.49%)^#^

Means within the same column that do not share a common superscript letter (a–c) differ significantly at *p* < 0.05, n = 6. Significant differences were found in CdCl_2_ vs. control (LDL and TG, *p* < 0.0001; HDL, *p* = 0.00216), AgNPs vs. CdCl_2_ (LDL and TG, *p* < 0.0001; HDL, *p* = 0.0025), and AgNPs + CdCl_2_ vs. CdCl_2_ (LDL and TG, *p* < 0.0001; HDL, *p* = 0.00457).

^#^Percentage of change between brackets = [(M_AgNPs+CdCl2_−M_CdCl2)_/M_CdCl2_] × 100.

AgNP-treated rats demonstrated normal levels of testicular hormones, close to the values in the control group, indicating non-toxic effects of the extract on the testes. However, the treatment of CdCl_2_-intoxicated rats with synthesized AgNPs from extracts induced a significant decrease in the levels of LDL and TG by 52.74% and 53.49%, respectively (*p* < 0.0001), coupled with a significant increase in the HDL level by 114.57%, compared to those in the CdCl_2_ group (*p* < 0.01), suggesting the hypolipidemic effect of the synthesized AgNPs of combined extracts toward the toxicity of CdCl_2_.

#### 3.2.5 Effect of synthesized AgNPs on the antioxidant biomarkers

The data presented in Table 5 demonstrated that the TAC and MDA levels were not significantly changed (*p* > 0.05) in rats that received AgNPs only, compared with the healthy control group. Meanwhile, TAC was significantly decreased (by 5.78-fold, *p* < 0.01), with an increase of MDA (by 1.77-fold, *p* < 0.001) in CdCl_2_ rats, compared with the healthy control group. The treatment of CdCl_2_ rats with synthesized AgNPs significantly increased TAC by 325.93% (*p* < 0.01) and reduced MDA by 39.49%, compared with those in the CdCl_2_ group (*p* < 0.05).

**TABLE 5:** Effect of different treatments on the antioxidant biomarkers.

Group	TAC (mM/L)	MDA (nmol/L)
Control	3.12 ± 0.46^a^	8.67 ± 1.95^b^
AgNP	3.16 ± 1.38^a^	8.78 ± 3.69^b^
CdCl_2_	0.54 ± 0.12^b^	15.32 ± 3.33^a^
AgNP + CdCl_2_	2.30 ± 0.72^a^ (+325.93%)^#^	9.27 ± 2.62^b^ (−39.49%)^#^

Values expressed as the mean ± SD of six replicates. Means within the same column that do not share a common superscript letter (a–b) differ significantly at *p* < 0.05, n = 6. Significant differences were detected in CdCl_2_ vs. control (TAC, *p* = 0.00475; MDA, *p* = 0.00067), AgNPs vs. CdCl_2_ (TAC, *p* = 0.00554; MDA, *p* = 5.73E-04), and AgNPs + CdCl_2_ vs. CdCl_2_ (TAC, *p* = 0.01042; MDA, *p* = 0.01653).

^#^Percentage of change between brackets = [(M_AgNPs+CdCl2_−M_CdCl2)_/M_CdCl2_] × 100.

### 3.3 Histopathological studies

#### 3.3.1 Effects of synthesized AgNPs on the liver tissues

**FIGURE 3:**
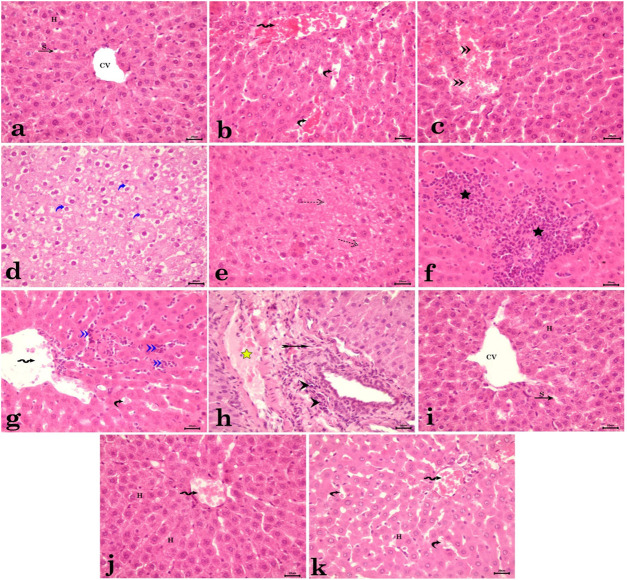
Photomicrograph of hepatic tissue sections stained with H&E. **(a)** Control group showing normal histological structure of the liver with central vein (CV), sinusoids (S), and multiple radiating plates of hepatocytes (H). **(b–h)** CdCl_2_-administered rats. **(b)** Congested central veins (wavy arrow) and sinusoids (curved arrow). **(c)** Focal hemorrhages (double arrowhead). **(d)** Diffused vacuolar degeneration of hepatocytes (blue curved arrow). **(e)** Areas of focal coagulative necrosis of hepatocytes (dotted arrow). **(f)** Focal infiltration of inflammatory cells (star). **(g)** Diffused infiltration of inflammatory cells within hepatic parenchyma, dilated central vein (wavy arrow), and congested blood sinusoids (curved arrow). **(h)** Congested portal blood vessel (yellow star) and connective tissue proliferation around the bile ductules (forked arrow) infiltrated with periductular inflammatory cells (arrowhead). **(i)** The rats treated with bio-synthesized AgNPs showed normal histological structure of the liver with central veins (CV) and multiple radiating plates of hepatocytes (H) and sinusoids (S) in between. **(j,k)** Group administered bio-synthesized AgNPs along with CdCl_2,_ showing normal histological architecture of the hepatic parenchyma with minimal histopathological changes in some examined cases, represented by slight congestion of the central vein (wavy arrow) and sinusoids (curved arrow).

Histopathological studies on the control group showed normal histological structure of the liver, with a central vein, sinusoids, and multiple radiating plates of hepatocytes (Figure 3a). However, in CdCl_2_-treated rats, there were marked structural alterations in the hepatic parenchyma in the form of congested central veins (Figure 3b) and sinusoids, along with focal hemorrhages (Figure 3c). Additionally, there was diffuse vacuolar degeneration of hepatocytes (Figure 3d), along with areas of focal coagulative necrosis (Figure 3e). Furthermore, the hepatic parenchyma exhibited focal (Figure 3f) and diffuse (Figure 3g) infiltration of inflammatory cells. Portal areas showed congested blood vessels and connective tissue proliferation around the bile ductules, infiltrated with periductular inflammatory cells (Figure 3h). The rats treated with bio-synthesized AgNPs only demonstrated normal histological structure of the liver, with central veins and multiple radiating plates of hepatocytes (Figure 3i). Simultaneous administration of bio-synthesized AgNPs along with CdCl_2_ revealed normal histological architecture of the hepatic parenchyma, with minimal histopathological changes in some examined cases, represented by slight congestion of the central vein and sinusoids (Figures 3j,k).

#### 3.3.2 Effects of synthesized AgNPs on kidney tissues

**FIGURE 4:**
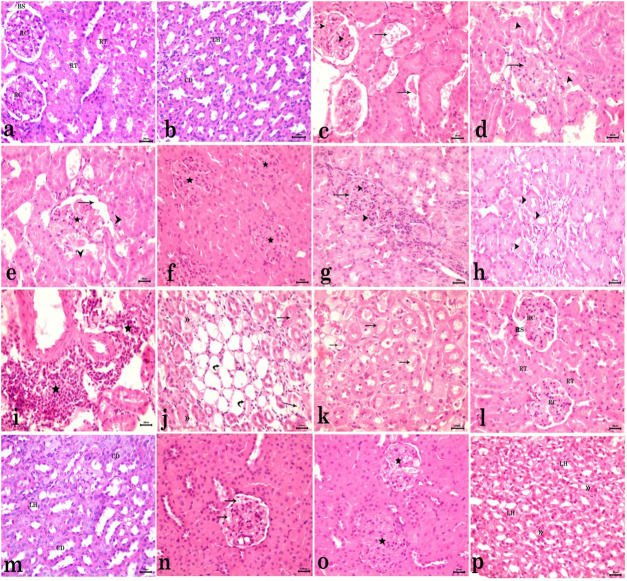
Photomicrograph of kidney tissue sections stained with H&E. **(a,b)** Normal control group. **(a)** Normal histological structure of the renal cortex with normal Malpighian corpuscles (RC), Bowman’s space (BS), and renal tubules (RT). **(b)** Normal medulla with normal collecting ducts (CD) and loop of Henle (LH). **(c–k)** CdCl_2_-administered group. **(c)** Marked congestion of glomerular tufts and interstitial blood vessels. **(d)** Some distorted renal corpuscles with damaged Bowman’s capsule (arrow) and necrosed renal tubular epithelium (arrowhead). **(e)** Shrinkage of the glomerulus (star) with slightly dilated Bowman’s space (arrow) and necrosed renal tubules (arrowhead). **(f)** Swollen glomeruli with increased cellularity and complete obliteration of the Bowman’s space (star). **(g)** Swollen glomeruli (arrow) with increased cellularity (arrowhead) and complete obliteration of the Bowman’s space. **(h)** Coagulative necrosis of renal tubules (arrowhead). **(i)** Perivascular infiltration of inflammatory cells (star). **(j)** Medulla with vascular congestion (double arrowhead), necrosis (arrow), and sloughing (curved arrow) of the lining epithelium. **(k)** Epithelium of the loop of Henle with intraluminal eosinophilic proteinaceous material (arrow). **(l,m)** AgNPs-treated rats. **(l)** Normal structure of the renal cortex with renal corpuscle (RC), Bowman’s space (BS), and renal tubules (RTs). **(m)** Medulla with normal collecting ducts (CDs) and loop of Henle (LH). **(n–p)** Rats co-administered CdCl_2_ and AgNPs. **(n)** Normal histological structure of the renal cortex with normal renal corpuscles with congested glomeruli in some renal corpuscles (arrow). **(o)** Swollen other glomeruli with hypercellularity and obstructed Bowman’s space (star). **(p)** Partially distorted loop of Henle (LH) in the medulla, accompanied by mild congestion of interstitial blood vessels (double arrowhead).

Light microscopic examination of the kidney tissues of control rats showed a normal histological structure of the renal cortex, with normal Malpighian renal corpuscles, proximal convoluted tubules, and distal convoluted tubules. Regarding the renal medulla of the control group, it showed normal collecting ducts and loop of Henle (Figures 4a,b). On the other hand, the CdCl_2_-administered group showed abnormal histological structure of the renal cortex and medulla. The cortex revealed marked congestion of glomerular tufts and interstitial blood vessels in most examined sections of this group (Figure 4c). Some renal corpuscles were distorted with damaged Bowman’s capsules (Figure 4d), shrunken with slightly dilated Bowman’s spaces (Figure 4e), or swollen with increased cellularity and complete obliteration of the Bowman’s space (Figures 4f,g). Renal tubules showed coagulative necrosis of the lining epithelium (Figure 4h). There was perivascular infiltration of inflammatory cells (Figure 4i). In the medulla, remarkable congestion of interstitial blood vessels, necrosis and sloughing of the tubular lining epithelium, and intraluminal eosinophilic proteinaceous material were observed (Figures 4j,k). In AgNP-treated rats, there was a normal structure of both the renal cortex and medulla (Figures 4l,m). Meanwhile, rats co-administered CdCl_2_ and AgNPs displayed a normal histological structure of the renal cortex with normal renal corpuscles. In addition, congested glomeruli of some renal corpuscles (Figure 4n) were observed. Other glomeruli were swollen with hypercellularity and obstructed Bowman’s space (Figure 4o). Moreover, the renal medulla of this group revealed partially distorted ducts and loop of Henle, accompanied by mild congestion of interstitial blood vessels (Figure 4p).

#### 3.3.3 Effects of synthesized AgNPs on testicular tissues

**FIGURE 5:**
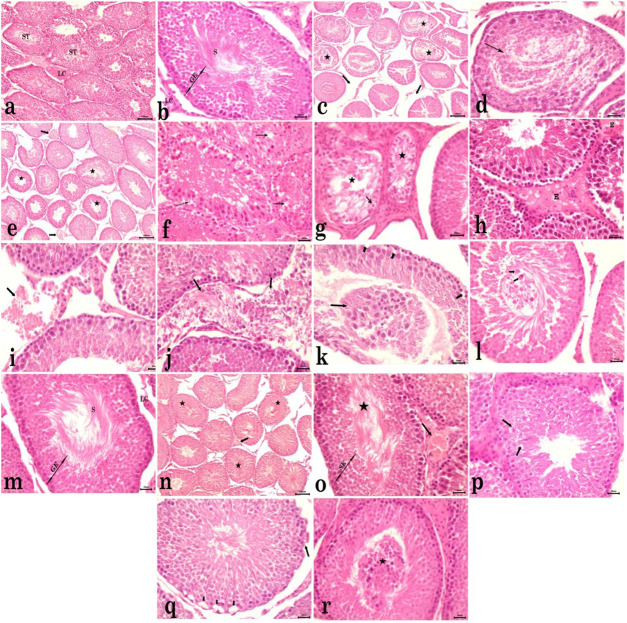
Photomicrographs of testicular tissue sections stained with H&E. **(a,b)** Control rats showing normal histomorphological criteria of seminiferous tubules (STs) with normal germinal epithelium (GE), spermatozoa (S), and Leydig cells (LCs). **(c-l)** Rats received CdCl_2_. **(c)** Necrosed germ cells in most seminiferous tubules (star) and disintegration of the interstitial tissues (arrow). **(d)** Necrosed tissue in the lumen of the seminiferous tubule with only a few layers of spermatogenic epithelium, spermatogonia, and primary spermatocytes. **(e)** Atrophied seminiferous tubules with irregular contour (star) and congestion of interstitial blood vessels (notched arrow). **(f)** Cytoplasmic vacuolation of germinal epithelium (arrow). **(g)** Degenerated tubules (star) with the appearance of only a few vacuolated germ cells, lining the degenerated tubules (arrow), reduced spermatogenesis, and the absence of spermatozoa in the lumen. **(h)** Interstitial edema (E). **(i)** Interstitial hemorrhages (arrow). **(j)** Seminiferous tubule with disrupted basement membrane, along with complete loss of normal architecture (arrow). **(k)** Cytoplasmic vacuolation of spermatogonia (notched arrow) and exfoliated germinal epithelium into the lumen of the seminiferous tubule (arrow). **(l)** Spermatid giant cells in the tubular lumen (arrow). **(m)** Testes of rats received only AgNPs showed normal architecture with normal seminiferous tubules (STs), germinal epithelium (GE), and interstitial Leydig cells (LCs). (n–r) Rats co-treated with CdCl_2_ and AgNPs. **(n)** Normal architecture of almost entire seminiferous tubules (star) and mild congestion of testicular blood vessel (arrow). **(o)** Normal histological criteria of seminiferous tubules with active spermatogenesis and accumulation of spermatozoa in lumen (star) and congestion of interstitial blood vessels (arrow). **(p)** Mild testicular degeneration characterized by cytoplasmic vacuolation of a few germ cells (arrow). **(q)** Mild vacuolation of spermatogonia (arrow) and undulant basement membrane (notched arrow). **(r)** Exfoliated germ cells into the lumen of the seminiferous tubule (star).

The testicular tissue sections from control rats exhibited normal histomorphological criteria of seminiferous tubules with active spermatogenesis (Figures 5a,b). However, rats that received CdCl_2_ showed necrosed germ cells in most seminiferous tubules and disintegration of the interstitial tissues (Figures 5c,d). Seminiferous tubules in most examined sections were atrophied with irregular contours and showed degenerative changes (Figure 5e). Some of the atrophied tubules displayed slight vacuolation of germ cells (Figure 5f), whereas most tubules showed distinct germinal epithelium vacuolar degeneration, leaving only one or two layers with reduced spermatogenesis and absence of spermatozoa in the lumen (Figure 5g). Moreover, congestion of interstitial blood vessels, interstitial edema (Figure 5h), and interstitial hemorrhage (Figure 5i) were evident. Seminiferous tubules with disrupted basement membranes, along with complete loss of normal architecture, were also detected (Figure 5j). Furthermore, many tubules showed the presence of exfoliated germinal epithelium and spermatid giant cells in the lumen of the seminiferous tubule (Figures 5k,l). The testes of the group that received only AgNPs showed normal architecture, with normal spermatogenesis in seminiferous tubules (Figure 5m). Meanwhile, the testes of rats co-treated with CdCl_2_ and AgNPs demonstrated normal architecture of almost the entire seminiferous tubules, with normal histological criteria, active spermatogenesis, and accumulation of spermatozoa in the lumen. However, slight lesions were observed, including mild congestion of interstitial blood vessels, mild cytoplasmic vacuolation of a few germ cells, and exfoliated germ cells into the lumen of the seminiferous tubule (Figures 5n–r).

## 4 Discussion

In the present study, we shed light on the modulatory effects of synthesized AgNPs from a combination of three plant extracts, namely, *P. crispum*, *Z. mays silk*, and *Acacia senegal*, on the hepato-renal and testicular changes induced by CdCl_2_ in male rats. Furthermore, the study investigated any possible side effects caused by AgNP administration in normal rats.

The obtained uniform size of AgNPs, as revealed by DLS and SEM analysis, highlights their potential as a highly effective nanomedicine for medical applications that require precise control of particle size and morphology. Additionally, negative zeta-potentials and low PDIs indicate an extremely stable colloidal system, highlighting AgNPs' potential for long-term stability for sustained activity in practical applications. The synthesis of AgNPs using a mixture of different extracts was established as the color of AgNO_3_ changed to a reddish color (Safaepour et al., 2009). Thus, the natural extract is also used to reduce Ag^+^ ions to Ag^0^, and a color conversion signaling is due to the efficacious formation of AgNPs. The results were in agreement with those reported by Moraes et al. (2010), demonstrating that the size, PDI, and charges of NPs are factors that reveal the stability of NPs. Furthermore, the obtained PDI is an indicative factor of the homogeneity of particles (Vieville et al., 2011). Notably, the particle size obtained from DLS was larger than that observed with SEM. This discrepancy is commonly reported as DLS measures the hydrodynamic diameter, which includes the metallic core and the surrounding hydrated shell formed by phenolic or flavonoid compounds, whereas SEM provides only the solid metallic core image. In addition, the SEM images revealed structural features influenced by the vacuum environment and surface coatings around the Ag^0^ core, which can affect apparent particle density (Abdellatif et al., 2023). Complementary TEM analysis confirmed the spherical morphology of the synthesized AgNPs, with particle sizes ranging between 5.28 and 21.47 nm, in agreement with earlier studies (Helmy et al., 2020b).

The results showed that the administration of AgNPs of a combined extract did not significantly exert any harmful effects on the liver functions, kidney functions, and testicular hormones of healthy animals, supported by histopathological studies. The liver is considered the key organ in metabolism, detoxification, and secretory functions in the body (Ukwuani et al., 2012). The expression of toxicity of xenobiotics is usually determined biochemically by monitoring plasma enzymes (Ukwuani et al., 2012). Enzymes such as AST and ALT are mainly localized in the cytoplasm, and any damage in hepatic cells may result in an alteration in the serum level (Halim et al., 1997). Thus, the changes in the activity of these marker enzymes in liver tissues could reflect the state of hepatotoxicity (Vinitha et al., 1995). High activities of serum aminotransferases (AST and ALT) are common markers of hepatic damage and have been reported more frequently in cadmium toxicity (Genchi et al., 2020). In the present study on CdCl_2_-induced toxicity, our results clearly indicated that the activities of AST, ALT, and ALP enzymes in CdCl_2_-intoxicated rats were significantly increased, likely due to cadmium binding to sulfhydryl groups, leading to mitochondrial permeability transition and mitochondrial dysfunction, with severe oxidative stress (Matović et al., 2015). The co-administration of AgNPs of the combined extracts showed significant hepatoprotective activity in rats receiving CdCl_2_, manifested by the restoration of normal histological structure of the liver with a central vein, sinusoids, and multiple radiating plates of hepatocytes, aligned with a decrease in elevated activities of ALT, AST, and ALP in intoxicated rats. In accordance with our results, parsley was reported to improve the hepatic degenerative changes in STZ-induced diabetic rats at a dose of 2 g/kg for 28 days (Ozsoy-Sacan et al., 2006), dexamethasone-induced hepatotoxicity (Khalil et al., 2015), and CCl_4_-induced liver damage. In addition, the gum acacia extract reversed the hepatic damage induced by gentamycin and enhanced the regenerative and reparative capacity of the liver for 8 days (Alubaidy, 2013). In addition, the gum acacia extract was reported to protect against hepatic oxidative stress in an alloxan-induced diabetic rat model (Ahmed et al., 2015) and acetaminophen-induced hepatotoxicity in mice (Gamal El-Din et al., 2003). Mechanistically, another study reported that the combination of selenium-enriched yeast and gum acacia diminished oxidative liver damage by inhibiting the expression of caspase-3 and pro-inflammatory genes in CCl_4_-intoxicated rats (Hamid et al., 2021). Furthermore, the corn silk infusion (200 mg/kg) was found to reduce ALP by 18.74% in a rat model of CCl_4_-induced hepatotoxicity (Ramadani et al., 2020). Moreover, it exhibited hepatoprotective properties against dose-induced injury of ecstasy (MDMA) in the isolated rat liver perfusion system (Karami et al., 2013). The histopathological alterations observed in the liver section also support our biochemical findings.

Biochemical indices such as the secretory substances of the kidney can be used as markers for assessing the normal functional capacities of different parts of the nephrons (Yakubu et al., 2003). These parameters of organ function, if altered, would impair the normal functioning of the organs (Abolaji et al., 2007). Similarly, the serum concentrations of urea and creatinine could provide insights into the effect of the plant extract on the tubular and glomerular parts of the kidney. In our study, the synthesized AgNPs had no harmful effect on the kidney functions of healthy animals, suggesting the normal functioning of the nephrons at the tubular and glomerular levels and the safety of plant extracts on renal functions. However, the administration of synthesized AgNPs of combined extracts improved the kidney function of CdCl_2_-intoxicated rats by decreasing the serum levels of creatinine and urea; thus, they could counter the renal changes associated with cadmium toxicity. Hence, the combined extracts have beneficial effects on renal functions. Mechanistically, the extracts are rich in many phenolic compounds, as mentioned in the chemical review; these compounds have multiple hydroxyl groups that act as metal chelators and can coordinate with Cd^2+^ (Emamverdian et al., 2015). Our investigation agreed with previously published studies (Elkhadragy et al., 2018; Gabr et al., 2019). Regarding the protective effects of the nanoparticles on the kidney tissues confirmed by histopathological studies, a study was conducted on the combination of *P. crispum*, *Z. mays*, and *Acacia senegal* to evaluate their nephroprotective activity in mice injected with amikacin (1.2 g/kg) (Helmy et al., 2020a). The combination extracts significantly inhibited BCL-2-associated X protein (BAX) and cytosolic cathepsin D, along with the upregulation of lysosomal-associated membrane protein-1 (LAMP-1) and nuclear transcription factor (TFEB) levels and the modulation of G-protein-coupled receptors (GPRs) that activated lysosome biogenesis, overcoming the adverse effects of amikacin on kidney tissues. The histopathological alterations observed in the kidney section also support our biochemical findings.

Concerning the effects of Cd on the male reproductive system, the testis is very sensitive to Cd, which intensely induced testicular damage and irreversible infertility (Oguzturk et al., 2012). Furthermore, the testicular oxidative stress induced by Cd resulted in atrophy of the testis, early death of germ cells at the development stage, irreversible cell damage in the testicular tissues, and deterioration of sperm characteristics (El-Demerdash et al., 2004). Some studies suggested that exposure to Cd decreases testicular sperm count and exaggerates sperm abnormalities, which could be associated with androgen changes and low levels of testosterone, LH, and FSH (Biswas et al., 2001). The histopathological testicular lesion in cadmium-intoxicated rats demonstrates the necrosed germ cells in the lumens of most seminiferous tubules, disintegration of the interstitial tissues, atrophied seminiferous tubules with irregular contour, congestion of interstitial blood vessels, and interstitial edema and hemorrhage, along with reduced spermatogenesis and absence of spermatozoa in the lumen (De Souza Predes et al., 2010; El-Shahat et al., 2009). In addition, the reduced testosterone level could be attributed to the downregulation of testicular LH receptors and production of cyclic adenosine monophosphate (Gunnarsson et al., 2003). On the other hand, this reduced level could be one of the consequences of decreased viability of Leydig cells due to the necrobiotic effects of Cd (Yang et al., 2003). Our findings, which are consistent with previous reports (De Souza Predes et al., 2010; El-Shahat et al., 2009), confirm the toxic properties of Cd on the testis of rats. Interestingly, the therapeutic intervention with AgNPs from combined extracts at a dose of 200 mg/kg b.w., administered 90 min following Cd injection, successfully attenuated the deleterious reproductive effects of Cd, restoring the normal architecture of testicular tissue with concomitant enhancements in the levels of testosterone, LH, and FSH. The observed therapeutic potency of AgNPs might be due to several contributing factors, primarily including the hormone-mediated effects elicited through their content of gonadotropin-like substances or steroidal components that act as gonad-stimulating compounds, improving male fertility and maintaining normal serum levels of testosterone (El-Neweshy et al., 2013). Furthermore, AgNPs may ameliorate Cd-induced oxidative stress in the testicular tissues, as evidenced by the renewal of spermatogenesis in the seminiferous tubules and normalization of the testicular histoarchitecture. The antioxidant properties of AgNPs may, therefore, reasonably explain their beneficial role in obviating the adverse effects of Cd on testicular tissues. Furthermore, a study of *P. crispum* in 108 male mice was found to improve the serum testosterone levels, reduce chromosomal aberrations, and enhance sperm count and motility in ZEN-induced clastogenicity (Hassan and Abdel-Wahhab, 2006). The treatment of hookah smoke-exposed rats with gum acacia mitigated the adverse actions on the reproductive system (testosterone, estradiol, luteinizing hormone, and androgen-binding protein) in male mice by inhibiting inflammation, oxidative stress, and nitrosative stress via a mechanism involving Nrf2 and reducing StAR expression (Ali et al., 2020). The histopathological alterations observed in the testis section also support our biochemical findings.

Our results on cadmium treatment in rats clearly exhibit alterations in the serum lipid profile. The increase in Cd burden in the body upsurges the risk of dyslipidemia, mainly due to the low HDL chol. level and the high ratio of triglycerides to HDL-chol (Kantola et al., 1998). This may be due to changes in the gene expression of hepatic enzymes such as hydroxy-methylglutaryl-CoA (HMG-CoA) reductase, which, in turn, depresses LDL-receptor gene expression and elevates the hepatic synthesis of triglyceride, with a reduced rate of clearance of triglyceride-rich lipoproteins (Afolabi et al., 2012; Mantur et al., 2014). In the present study, the significant improvement in the lipid profile of Cd-intoxicated rats treated concomitantly with synthesized AgNPs agrees with other studies reporting that parsley can protect against serum lipid abnormalities in hypercholesterolemic rats (Kaddam et al., 2019; Oyeyemi et al., 2018). No changes in the lipid profile were observed in rats after AgNP supplementation alone compared to the control, possibly due to the abilities of the synthesized AgNPs to maintain all the lipid profile parameters within the normal range.

Basically, cadmium accumulates primarily in the kidney and liver, with an estimated clearance half-life of 25 years; these two organs are the critical targets for acute cadmium toxicity; approximately 60% of the entire cadmium that enters the body is deposited in the liver and kidneys, while the remaining 40% is distributed throughout the body (Bernhoft, 2013; Zhai et al., 2013). One mechanism of cadmium-induced liver damage is its interaction with essential sub-cellular sites, such as mitochondria, peroxisomes, and microsomes, exaggerating ROS generation and lipid peroxidation expressed as MDA (Matović et al., 2011).

In addition, cadmium is capable of indirectly eliciting oxidative damage to the liver by depleting cellular antioxidant levels, particularly GSH, and protein-bound sulfhydryl groups, which promotes the generation of ROS, such as superoxide ion, hydroxyl radicals, and hydrogen peroxide (El-Refaiy and Eissa, 2013; Wang et al., 2015). Cadmium-induced liver damage is believed to be related to the interactions of these ROS with cellular biomolecules, which alter numerous cellular functions, such as enzyme activities, gene expression, and DNA repair mechanisms, along with signal transduction, and cause a shift in the overall cell redox state. In addition, cadmium competes with essential metals, such as zinc, selenium, copper, and calcium, thereby interfering with various cellular processes, such as metal membrane transport and energy metabolism (Arroyo et al., 2012). Documented scientific evidence shows that Cd interacts with biomolecules and initiates lipid peroxidation, leading to oxidative stress associated with various cellular damage (Nazima et al., 2015). A direct relationship exists between the level of tissue impairment and the level of produced MDA; the level of MDA can be utilized as an index of *in vivo* peroxidative damage and the assessment of the vulnerability of tissues to oxidative stress (Ayala et al., 2014). Therefore, the decreased TAC associated with the elevated MDA level in the cadmium-treated rats in our study is evidence of increased membrane lipid peroxidation; this observation is in agreement with earlier reports (Asagba et al., 2007; Ding et al., 2013). The treatment of Cd-exposed rats with synthesized AgNPs of combined extracts could enhance TAC and reduce MDA levels, suggesting the ability of AgNPs of combined extracts to mitigate Cd-induced lipid peroxidation. Cadmium interacts with these cellular biomolecules, depletes endogenous reduced GSH and protein-bound sulfhydryl groups, and promotes the overproduction of ROS such as hydrogen peroxide, hydroxyl radicals, and superoxide (Shukla and Kumar 2009). The diminished level of liver GSH in Cd-treated rats leads to the reduced antioxidant defense system in maintaining an oxidant/antioxidant balance during cadmium toxicity. Furthermore, devastating oxidative alteration in enzymatic proteins and bio-membrane lipids by ROS resulted in a significant reduction in the cellular antioxidant defense system, such as SOD and CAT. Additionally, cadmium has been shown to directly inhibit SOD and CAT activities through Cd–enzyme interactions, resulting in the agitation of enzyme topography imperative for catalytic activity (Zhang et al., 2024). The treatment with AgNPs of combined extracts reversed these changes, signifying the antioxidant role of synthesized AgNPs. Several reports confirmed the antioxidant properties of *P. crispum* (Alagawany et al., 2024; Nouioura et al., 2024), *Z. mays* (Ali M. et al., 2024; Ali Q. et al., 2024), and *A. senegal* (El-Ratel et al., 2025; Kathan et al., 2025).

## 5 Conclusion

The administration of green-synthesized AgNPs derived from a combined extract of *P. crispum*, *Z. mays silk*, and *Acacia senegal* significantly ameliorated the liver and renal functions of CdCl_2_-intoxicated rats. Additionally, AgNPs remarkably restored testicular hormone levels, testosterone, LH, and FSH, indicating notable protection of male reproductive functions. The initial hormonal status may serve as a predictive biomarker for therapeutic responsiveness to treatment. Notably, AgNP administration showed no detectable toxicity in healthy rats, highlighting its biosafety in the tested setting. Collectively, these findings indicate that green-synthesized AgNPs from edible plant sources can serve as promising candidates to counteract environmental CdCl_2_ toxicity, primarily through antioxidant and free radical scavenging activity. Future studies should investigate long-term safety, real-world stability, and clinical or environmental efficacy to establish the utility and commercial applicability of plant-based AgNPs in mitigating heavy-metal toxicity.

## Data Availability

The original contributions presented in the study are included in the article/supplementary material; further inquiries can be directed to the corresponding author.
